# Effects of short‐term nitrogen addition on the recovery of alpine grassland in the Tianshan Mountains of Xinjiang, China

**DOI:** 10.1002/ece3.70329

**Published:** 2024-10-08

**Authors:** Juan Wang, Junjie Liu, Chao Liu, Xiaoyu Ding, Yonggang Ma, Jianjun Yang, Zhonglin Xu

**Affiliations:** ^1^ College of Ecology and Environment, Xinjiang University Urumqi China; ^2^ College of Geography and Remote Sensing Sciences, Xinjiang University Urumqi China; ^3^ Technology Innovation Center for Ecological Monitoring and Restoration of Desert‐Oasis, MNR Urumqi China; ^4^ Key Laboratory of Xinjiang Oasis Ecology, Ministry of Education (Xinjiang University) Urumqi China

**Keywords:** grassland restoration, plant biomass, plant diversity, short‐term nitrogen addition, soil properties

## Abstract

The restoration of alpine grasslands has garnered significant attention across various sectors. Historically, natural restoration has been the primary approach for grassland recovery, characterized by its prolonged duration. To expedite the recovery of degraded grasslands, it is essential to identify the limiting factors of restoration, enabling efficient and rapid recovery. Appropriate nitrogen (N) addition levels have been considered a potential strategy to enhance the recovery of grassland ecosystems and augment their ecological benefits. However, the effectiveness of N addition in alpine grassland restoration remains debated. This study investigated the impact of five N addition levels (CK: control [0 g/m^2^]; LN: low N [5 g/m^2^]; MN: medium N [10 g/m^2^]; HN: high N [15 g/m^2^]; SN: severe N [20 g/m^2^]) and two experimental approaches (N addition once per year [NPY] and three times per year [NTY] at the same dosages) on plant and soil properties and the maximum restoration capacity of alpine meadows. Our findings reveal three key insights: The level of N addition was the primary factor influencing aboveground plant biomass and coverage. Plant diversity decreased under the NTY regime and increased with NPY in the Bayinbruck grassland. N addition significantly altered soil properties, including pH, salinity, soil organic carbon (SOC), soil‐available phosphorus (AP), and soil total phosphorus (TP). Notably, soil TP, total nitrogen (TN), and AP substantially impacted plant community structure and diversity. Based on structural equation model (SEM) and analysis of variance (ANOVA), optimal grassland restoration was achieved with the HN (15 g/m^2^) treatment under NPY and the MN and HN (10 and 15 g/m^2^) treatments under NTY. Overall, our study offers crucial insights into the conservation, management, and restoration of grassland ecosystems on the Bayinbruck Plateau. It underscores the significance of N addition effects on plant communities, vegetation restoration, and soil properties.

## Graphical abstract

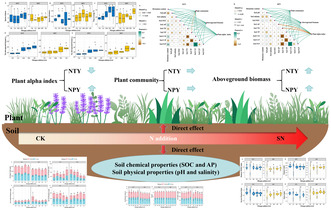

## 1 INTRODUCTION

Grasslands are among the world's most widely distributed terrestrial ecosystems, providing essential ecological and economic services such as soil and water conservation, livestock products, and biodiversity protection (Rodrigues et al., 2018; Sparrius & Kooijman, 2012). Globally, they occupy approximately 25% of the Earth's surface area (Fartyal et al., 2024; Padbhushan et al., 2020). Over the past 30 years, human activities and fossil fuel emissions have led to a continuous decline in the diversity and resilience of grassland ecosystems, severely threatening their service functions and regional ecological security (Chen et al., 2022; Sheng et al., 2022). Nitrogen (N) addition has been proposed as a rapid means to restore grassland productivity by replenishing soil nutrients, thereby contributing to the stability and healthy development of grassland ecosystems (Sparrius & Kooijman, 2012). However, the effectiveness of N addition in restoring degraded grasslands remains controversial (Vargová et al., 2020). While moderate N deposition can increase productivity, excessive N additions have been shown to decrease species richness in grassland ecosystems (Borgström et al., 2018; Liu et al., 2022, 2024). Thus, it is urgent to clarify the effective pathways of N addition for grassland restoration and to provide data supporting the maximum resilience of grassland ecosystems achievable in the short term.

Grassland restoration is characterized by increased productivity, biodiversity and community stability (Joshi & Garkoti, 2023; Yan et al., 2021). Constructive plant and soil nutrient cycling can also indicate enhanced grassland resilience (Prager et al., 2017). Theoretically, plant diversity determines the stability and functional characteristics of grassland ecosystems (Niu et al., 2018). Higher plant diversity can enhance grassland productivity and restoration capacity through species complementarity (Vargová et al., 2020). Previous researchers have used structural equation model (SEM) and Mantel tests to evaluate plant community‐soil quality stability and species turnover (Zhou et al., 2019). Although N additions can quickly restore productivity, N deposition can impact the structure and function of the entire ecosystem by increasing soil acidification (Li et al., 2020), reducing species diversity (Rodrigues et al., 2018; Sparrius & Kooijman, 2012), and altering community composition (Leimer et al., 2018). These studies have shown that plant diversity can influence the physical and chemical properties of soil through complementary and qualitative effects (Rodrigues et al., 2018; Sparrius & Kooijman, 2012). Additionally, soil fertility is often characterized by the stoichiometric ratios of C:N, C:P, and N:P (Fayiah et al., 2019), and appropriate fertilization has been shown to promote plant uptake of soil cations, thereby maintaining stable plant productivity (Prager et al., 2017). Ma et al. (2020) showed that in the process of vegetation restoration in different rocky desertification study areas, suitable species should be selected according to their different degradation levels and characteristics. Similarly, Yan et al. (2021) showed that strengthening grassland management would significantly promote the productivity increase of semi‐desert grassland. Based on previous studies, Is it then possible to speculate that aboveground productivity and plant community stability in grasslands tend to remain stable under long‐term protection or nitrogen fertilization restoration (Boudjabi & Chenchouni, 2022; Chen et al., 2022; Juan et al., 2023)? Consequently, we chose moderate N addition levels that have less influence on soil physical and chemical properties while enhancing grass productivity as the optimal strategy for grassland restoration (Hodapp et al., 2018; Liu et al., 2024).

The Bayinbruck alpine meadow, a vital grassland resource with high biodiversity, has become a priority area for global biodiversity conservation (Li et al., 2015). Over the past two decades, grazing has increased dramatically, with N deposition in the alpine meadows of Xinjiang Tianshan reaching approximately 8 kg N·ha^−2^·a^−1^ (Li et al., 2015). This has led to grassland degradation, soil erosion, and reduced vegetation diversity, seriously threatening the ecological security of the Bayinbruck alpine meadows (Zou et al., 2016). The physical and chemical properties of the soil provide a conducive environment and necessary elements for plant growth (Rao et al., 2021). Soil elements such as carbon (C), nitrogen (N), and phosphorus (P) significantly affect the growth and development of grassland vegetation communities (Rao et al., 2021). Previous studies have focused on the effects of grazing practices and long‐term N addition on grassland ecosystems, including changes in soil nutrients and alterations in plant communities (Li et al., 2015; Zou et al., 2016). However, few studies have investigated the effects of N addition on grassland vegetation restoration (Fayiah et al., 2019; Rodrigues et al., 2018). It is practicable that the vegetation diversity index and SEM and Mantel tests were used to explain the driving factors of grassland recovery (Ma et al., 2020). Therefore, further exploration is needed to understand the effects of N addition on grassland plant communities and soil physicochemical properties.

Based on the above reasoning, we hypothesize that the intensity of grassland restoration depends on the dose and frequency of nitrogen addition. If this is true, then grassland recovery responds differently to different nitrogen additions, especially control, low and high nitrogen. To validate the mechanism for assessing this, we conducted field experiments. At the Bayinbruck Alpine Grassland Experimental Site, field research data (including vegetation and soil data) were collected in 2021–2022 to explore the restorative capacity of short‐term N addition to grassland. Our study aims to answer the following questions: (1) What factors affect grassland recovery under different N addition treatments? (2) Does the ability of grassland to recover change with the frequency of N addition? (3) What level of N addition ensures maximum grass recovery in the short term? This paper aims to provide important insights into the conservation, management and restoration of grassland ecosystems in the Bayinbruck by answering the above three questions.

## 2 MATERIALS AND METHODS

### 2.1 Study area

A short‐term field nutrient addition experiment was conducted in an alpine grassland near Bayinbruk, Xinjiang, northwest China (83°70′ E, 42°88′ N). The average annual temperature is −4.7°C, with extreme monthly maximum and minimum temperatures ranging from 28°C to −48.1°C. Yearly maximum precipitation fluctuates between 216.8 mm and 361.8 mm during summer and autumn. There is no absolute frost‐free period, and the climate is typically alpine. The region experiences 160–180 days of snowpack, a 7‐month dry grass period, and has 4.7 m of permafrost. The soil is characterized as chestnut‐calcium and shallow. The Bayinbruk alpine grassland exhibits plant diversity, with the lowest recorded measurement being nine grass species per square meter (Juan et al., 2023).

### 2.2 Experimental design

**FIGURE 1:**
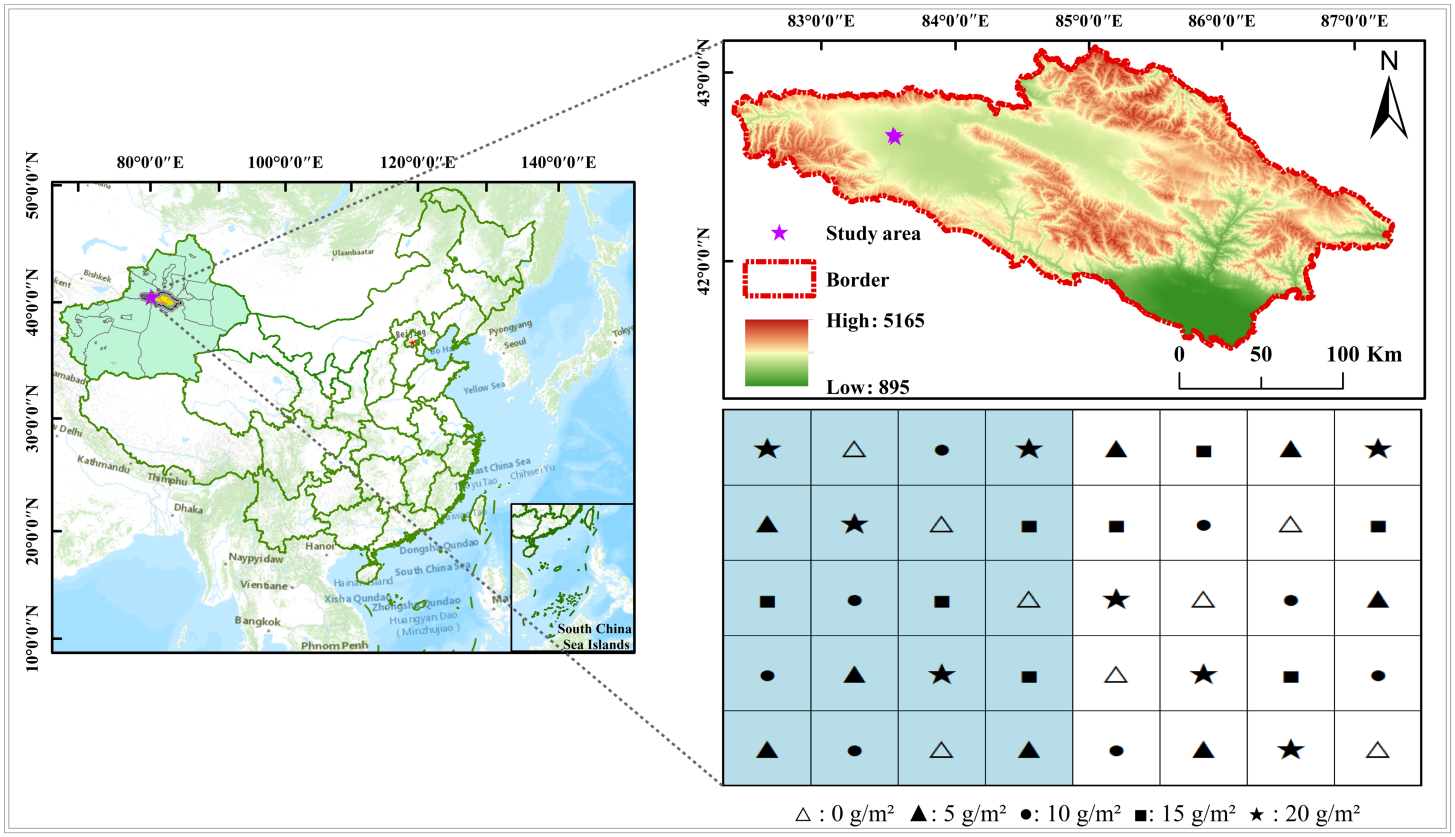
Study area map and sample distribution. The blue underlay represents NTY, and the white underlay is NPY, where △ indicates control (CK) (0 g/m^2^); ▲ indicates low N (LN) (5 g/m^2^); ● indicates medium N (MN) (10 g/m^2^); ■ indicates high N (HN) (15 g/m^2^); and ★ indicates severe N (SN) (20 g/m^2^).

The field experiment was conducted with NH₄NO₃ in 2020 in the alpine grassland of Bayinbruk, Xinjiang, northwest China. A completely random method was used to establish 40 plots (3 × 3 m) with a 1 m distance between them. The experiment consisted of two sets of N addition treatments, each with five different levels of N addition and four replications, resulting in a total of 10 treatments (as shown in Figure 1). The N addition experiment was divided into NPY (N added once per year) and NTY (N added three times per year) treatments. N was added at the same dosages in both NPY and NTY. For NTY, N addition was applied in April, May, and June by dividing the total N addition into three equal parts. For NPY, N addition was provided once in April.

### 2.3 Plant and soil methods

In late August 2021, vegetation surveys were conducted in randomly selected 0.5 × 0.5 m areas within the 40 large 3 × 3 m sample sites to record plant species abundance and richness. The total plant coverage was measured using the tip method with 16 square frames of 10 cm grid points (0.5 × 0.5 m). All plants within a 0.5 × 0.5 m square were cut flush with the ground and weighed for aboveground biomass, which was then dried to obtain the dry matter mass. Plant diversity indices were used to reflect species status and roles in the community. The Shannon‐Wiener index reflects the uncertainty of randomly selecting an individual plant in a community (Yuan et al., 2015). Simpson's index and the inverse Simpson index reflect species richness and evenness, while the Pielou index indicates species distribution within the community (Kinnebrew et al., 2019).

Shannon‐Wierner index:

$$H=-\sum_{i=1}^{S}\left(P_{i}\ln P_{i}\right)$$

where *S* is the number of species in the sample plot, and *P*_*i*_ is the importance value of the plant.

Simpson's index:

$$\mathrm{Cd}=1-\sum_{i=1}^{S}{\left(\frac{N_{i}}{N}\right)}^{2}$$

where *N*_*i*_ denotes the number of individuals of species *i*, and *N* represents the total number of individuals of all species.

Inverse Simpson Index:

$$\text{IS}=\frac{1}{\mathrm{Cd}}$$

Pielou index:

$$J=H/\ln\left(S\right)$$

After the vegetation survey, three soil samples were collected with a soil auger at depths of 0–10 cm and 10–20 cm from each sample plot and combined into two mixed samples, resulting in 80 soil samples. All plant and soil samples were transported to Xinjiang University for physical and chemical characterization (Table 1), including soil moisture content, pH, salinity, soil organic carbon (SOC), total nitrogen (TN), total phosphorus (TP), and available phosphorus (AP) (Fayiah et al., 2019). We list the measurements of soil physical and chemical properties in Table 1.

**TABLE 1:** The indicators for physical and chemical determination of soil.

Measurement indicators	Measurement methods
Soil moisture content	The drying method
Soil pH	The potentiometric method
Soil salinity	The potentiometric method
Soil organic carbon (SOC)	The K_2_Cr_2_O_7_‐FeSO_4_ titration method
Soil total nitrogen (TN)	The semi‐micro Kjeldahl method
Soil total phosphorus (TP)	The H_2_SO_4_‐HClO_4_ digestion method
Soil‐available phosphorus (AP)	The NaHCO_3_ method

### 2.4 Data analysis

Statistical analyses were conducted using IBM SPSS Statistics 25 and R (4.2.0). Analysis of variance (ANOVA) was performed to discern relationships between different variables, with significant differences at *p* < .05 indicated by different letters (Lu, Lu, et al., 2021). The Mantel test (explore the correlation between two matrices) was performed using the linkET package in R. Similarly, the piecewiseSEM package was employed for the structural equation model (SEM). SEM is a statistical method for analyzing relationships between variables based on a covariance matrix (Band et al., 2022; Borgström et al., 2018). This technique was used to discern the causal relationships between N addition, soil properties, plant communities, and plant productivity. The goodness of fit for the models was assessed using Fisher's test, AIC, BIC, and *p*‐values. Path coefficients were iteratively removed if they were ≥1 until all path coefficients were <1 and *p* > .05, indicating a good model fit (Hodapp et al., 2018).

## 3 RESULTS

### 3.1 Plant communities and diversity

**FIGURE 2:**
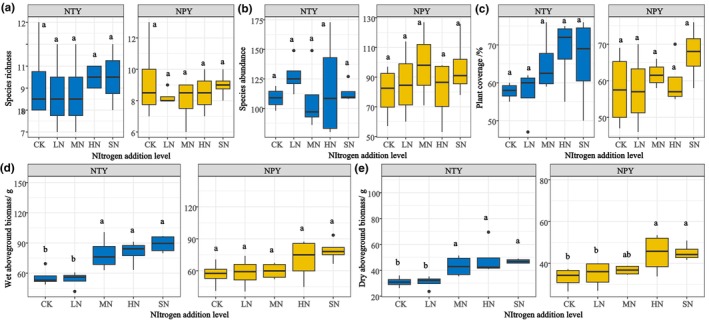
Effect of different N addition on plant communities (CK, Control [0 g/m^2^]; HN, High N [15 g/m^2^]; LN, Low N [5 g/m^2^]; MN, Medium N [10 g/m^2^]; SN, Severe N [20 g/m^2^]).

In summary, significant trends in species abundance, richness, cover, and aboveground biomass were observed at different levels of N addition between NTY and NPY (Figure 2). Species richness slightly increased by approximately 2.7% in HN and SN under NTY but declined by more than 10% in LN and HN under NPY. Similarly, changes in species richness across different levels of N addition were insignificant (*p* > .05) compared to the control (CK) (Figure 2a). As shown in Figure 2b, species abundance significantly increased in LN with NTY and MN with NPY, reaching 128 and 98.5, respectively. Plant community cover exceeded 60% in MN, HN, and SN treatments under NTY (65%, 68.5%, and 66%, respectively) and was highest in SN under NPY at 67.5% (Figure 2c). Trends in wet and dry weights of plants were similar under NTY and NPY (Figure 2d,e). Significant differences (*p* < .05) were observed for MN, HN, and SN (79, 80.7, and 88.9 g, respectively) compared to CK (56.1 g) in NTY, and for HN and SN (70.5 and 79 g, respectively) compared to CK (56.6 g) in NPY.

**FIGURE 3:**
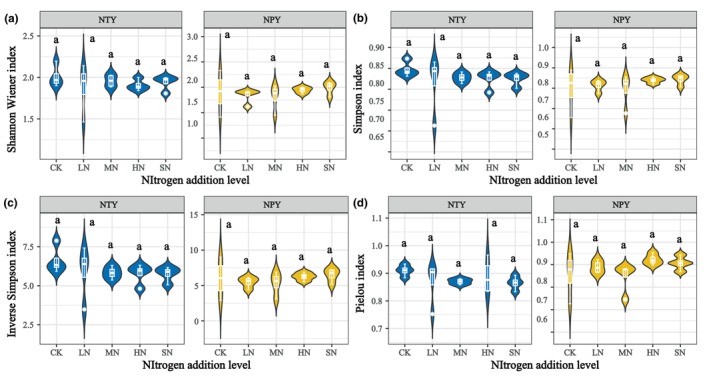
Effect of different N additions on plant diversity (CK, Control [0 g/m^2^]; HN, High N [15 g/m^2^]; LN, Low N [5 g/m^2^]; MN, Medium N [10 g/m^2^]; SN, Severe N [20 g/m^2^]).

The plant diversity indices were stable under both NTY and NPY treatments. The Shannon‐Wiener index showed a decreasing trend at 2.1, 1.91, 1.88, 1.96, and 1.94 for NTY and a decrease followed by an increase at 1.90, 1.95, 1.84, 1.82, and 1.99 for NPY (Figure 3a). The Simpson index (Figure 3b) changed insignificantly with different N additions. LN, MN, HN, and SN showed a continuous downward trend in NTY (3.79%, 2.34%, 2.72%, and 2.67%, respectively) and a constant increase in NPY (1.8%, 0.68%, 4.75%, and 5.2%, respectively) compared with CK. The inverse Simpson Index followed the same pattern as the Simpson Index (Figure 3c). The Pielou index (Figure 3d) showed a decreasing trend under NTY (4.67%, 4.58%, 2.46%, and 5.22%, respectively) and an increasing trend under NPY (3.54%, −1.4%, 6.99%, and 5.37%, respectively), compared with CK. The results indicate that N addition negatively affected plant diversity in NTY but had a positive effect in NPY (Figure 3).

### 3.2 Soil physical and chemistry properties

**FIGURE 4:**
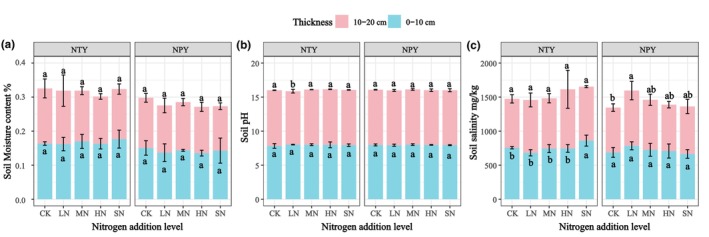
Effect of different N addition on soil physical properties (CK, Control [0 g/m^2^]; HN, High N [15 g/m^2^]; LN, Low N [5 g/m^2^]; MN, Medium N [10 g/m^2^]; SN, Severe N [20 g/m^2^]).

The effects of different treatments on the physical properties of the soil are shown in Figure 4. Differences among N addition treatments were insignificant (*p* > .05) for soil moisture content at 0–20 cm soil depths (Figure 4a). Soil pH was insignificant at 0–10 cm soil depths under NTY and NPY for all treatments (Figure 4b). However, in NTY, soil pH was significantly lower in the LN treatment at 10–20 cm soil depths compared to other N treatments. Soil salinity was significant (*p* < .05) at 0–10 cm soil depths but insignificant (*p* > .05) at 10–20 cm soil depths in NTY (Figure 4c). Differences in soil salinity between CK and LN treatments were significant at 10–20 cm soil depths in NPY (*p* < .05), while differences among MN, HN, and SN treatments were insignificant.

**FIGURE 5:**
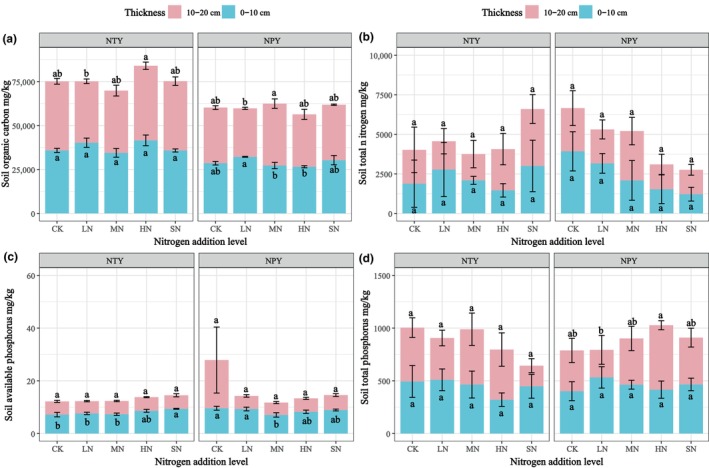
Effect of different N addition on soil chemical properties (CK, Control [0 g/m^2^]; HN, High N [15 g/m^2^]; LN, Low N [5 g/m^2^]; MN, Medium N [10 g/m^2^]; SN, Severe N [20 g/m^2^]).

Soil chemical properties (SOC, TP, and AP) were significant (*p* < .05) across different N addition treatments in NTY and NPY, as shown in Figure 5. The SOC (Figure 5a) increased at 0–10 cm soil depths in NTY and significantly changed at 10–20 cm soil depths (*p* < .05). SOC was also significantly different in NPY. The average SOC value was highest in LN at 0–10 cm soil depths and MN at 10–20 cm soil depths in NPY (*p* < .05). TN changes were insignificant for all N addition treatments (Figure 5b). TN showed an increasing trend in NTY, with the lowest values in HN at 0–10 cm and MN at 10–20 cm soil depths. In contrast, a linear decrease was observed in NPY, with mean values at 0–10 cm and 10–20 cm soil depths ranging from 3926.23 to 1214.25 mg/kg and 2734.2 to 1525.42 mg/kg, respectively. Soil available phosphorus (AP) at 0–10 cm soil depths in NTY increased from 7.16 mg/kg in CK to 9.37 mg/kg in SN and decreased from 9.58 mg/kg in CK to 7.04 mg/kg in MN in NPY (Figure 5c). TP at 0–10 cm soil depths showed a decreasing trend in NTY and a slowly increasing trend in NPY (Figure 5d). At 10–20 cm soil depths, TP decreased in NTY (CK, 510.7 mg/kg; HN, 475.22 mg/kg; LN, 396.45 mg/kg; MN, 524.29 mg/kg; SN, 195.46 mg/kg) and increased in NPY (CK, 386.58 mg/kg; HN, 611.1 mg/kg; LN, 261.07 mg/kg; MN, 438.72 mg/kg; SN, 444.07 mg/kg).

**FIGURE 6:**
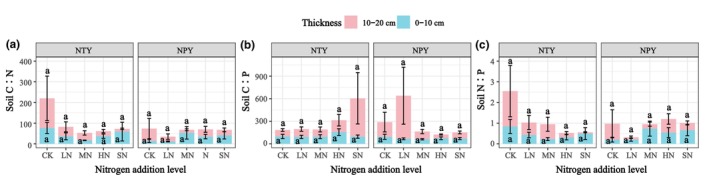
Effect of different N addition on soil stoichiometric ratios. Where CK, control (0 g/m^2^); HN, high N (15 g/m^2^); LN, low N (5 g/m^2^); MN, medium N (10 g/m^2^); SN, severe N (20 g/m^2^).

The effects of different N addition levels on soil stoichiometric ratios are shown in Figure 6. Differences in C:P, C:N and N:P ratios were insignificant (*p* > .05) under both NPY and NTY treatments. Compared with CK, the soil C:N ratios at 0–10 cm depth decreased by 52.48%, 77.94%, 51.5%, and 22.78% in the NTY treatment and increased by −17.88%, 281.3%, 150.68%, and 196.63% in the NPY treatment for LN, MN, HN, and SN, respectively. Similarly, at 10–20 cm soil depth, the C: N ratios decreased by 68.08%, 74.96%, 82.98%, and 90.67% in the NTY treatment and by 60.83%, 75.16%, 41.08%, and 56.03% in the NPY treatment compared with CK (Figure 6a). The C:P ratio at 0–10 cm soil depth showed an increasing trend under NTY with changes of −6.09%, −3.21%, +58.08%, and −2.13% compared with CK. In contrast, it showed a decreasing trend under NPY, with changes of 28.34%, 34.58%, 20.55%, and 25.13% compared with CK (Figure 6b). At 10–20 cm soil depth, the C:P ratios increased by 21.29%, 12.39%, 84.75%, and 494.65% in the NTY treatment and by +193.07%, −47.64%, −74.66%, and −58.1% in the NPY treatment compared with CK. The soil N:P ratio at 0–10 cm depth showed a decreasing trend under NTY by 49.43%, 72.44%, 62.32%, and 40.93% and an increasing trend under NPY by 2.5%, 262.65%, 168%, and 227.47% compared with CK (Figure 6c). At 10–20 cm soil depth, the N:P ratios showed a decreasing trend for all N addition treatments, with reductions of 64.54%, 58.05%, 87.71%, and 96.26% under NTY, and 85.38%, 71.96%, 14.18%, and 54.75% under NPY compared with CK.

### 3.3 Correlation analysis of plant communities and soil properties

**FIGURE 7:**
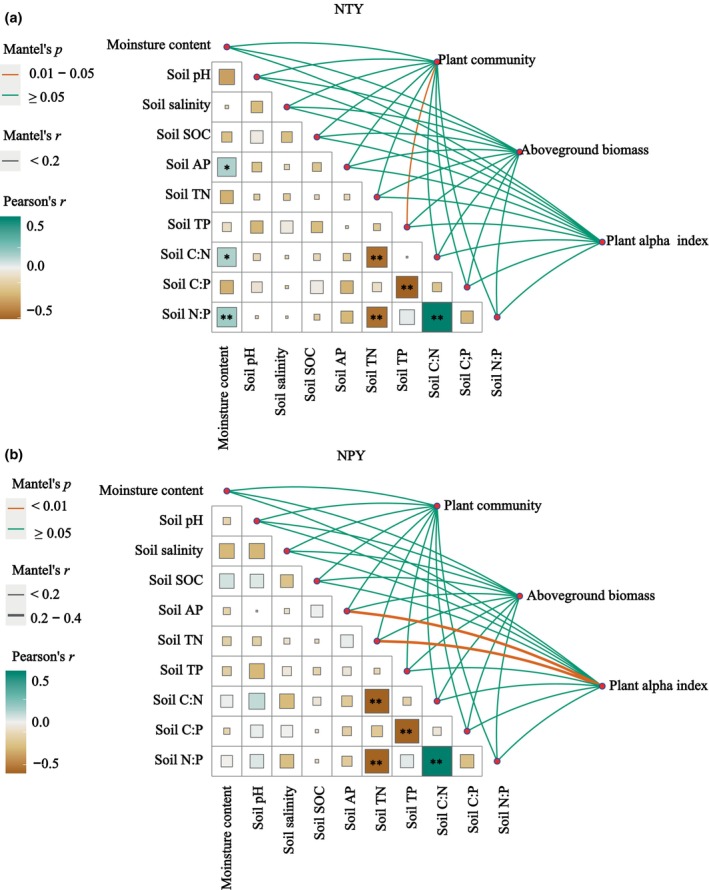
Environmental drivers of plant community and diversity (Pearson's correlation coefficients are shown in the semi‐matrices with a color gradient; ** indicates a significant correlation at the 0.01 level, and * indicates a significant correlation at the 0.05 level. The Mantel test was used to determine the correlation between the plant community and each soil factor. Edge widths correspond to Mantel's r statistic for distance correlation, and line colors indicate statistical significance).

In general, plant community metrics (species abundance, richness, and coverage) were more influenced by soil TP (*p* < .05) than by other soil factors (Figure 7a). Aboveground biomass (wet and dry) and plant alpha indices (Shannon‐Wiener index, Simpson index, inverse Simpson index, and Pielou index) were insignificantly influenced by soil properties. Correlations among soil physical properties, such as moisture content, salinity, and pH, were weak in NTY. However, soil moisture content was significantly positively correlated with soil AP, N:P, and C:N ratios (*p* < .05), and with soil N:P ratios (*p* < .01). Plant community diversity was significantly associated with soil AP and TP in a single application, whereas other factors were independent. Soil N:P, C:P, and C:N ratios were significantly negatively correlated (*p* < .01). As shown in Figure 7b, soil TN and AP influenced the plant alpha index more than other soil factors (*p* < .01). The plant community and aboveground biomass were not significantly correlated with soil properties (*p* > .05). Soil TN, TP, and C:N ratios were significantly associated with soil C:P and N:P ratios (*p* < .01).

### 3.4 Effects and pathway analyses

**FIGURE 8:**
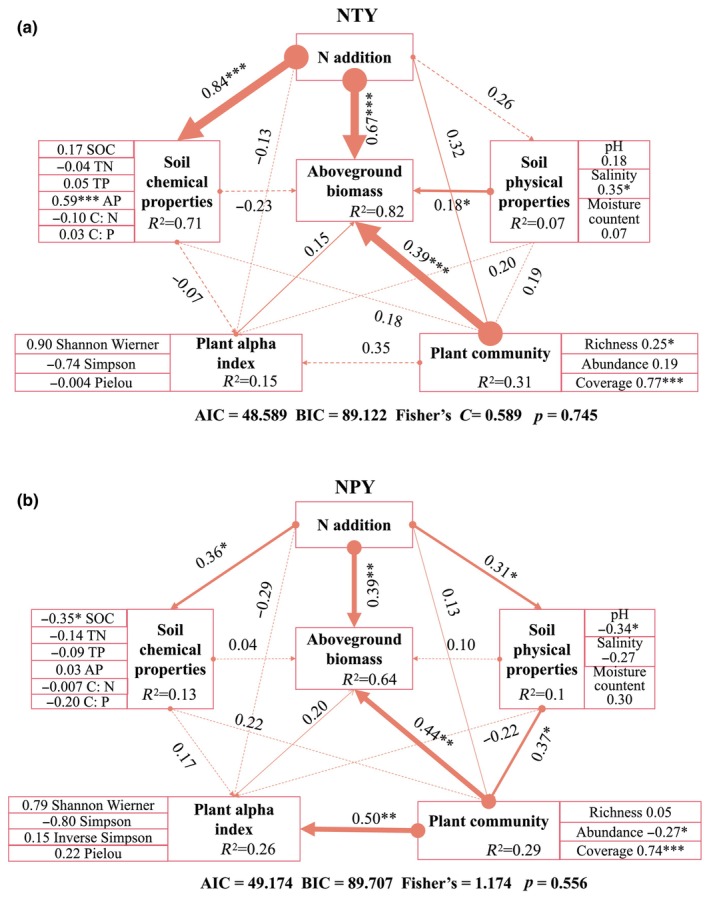
Model construction of plant community structure and soil properties (SEM shows the direct and indirect effects of two sets of N addition experiments on soil physical properties, soil chemical properties, plant alpha index, plant community, and aboveground biomass, as well as the standardized total effects obtained from SEM. Positive and negative relationships are found among consecutive arrow variables, the width of arrows plotted against the significance of path coefficients, and the level of value: ***p < .001, **p < .01, *p < .05).

The structural equation model (SEM) showed direct or indirect relationships between plant diversity, plant community, aboveground plant biomass, and soil properties under the two N‐addition experiments (Figure 8). The *R* ^2^ values of the N addition affecting soil chemical properties, plant community and aboveground biomass were 0.71, 0.31 and 0.82 in the NTY. The *R* ^2^ values of the N addition affecting plant alpha index, plant community and aboveground biomass were 0.26, 0.29 and 0.64 in the NPY. The NTY (Figure 8a) significantly affected soil chemical properties (soil AP) and aboveground plant biomass (*p* < .001), with pathway coefficients of 0.70 and 0.84, respectively. Similarly, aboveground plant biomass was significantly affected by soil physical properties (salinity: 0.35) and plant characteristics (species abundance and cover: 0.25 and 0.77, respectively), with pathway coefficients of 0.18 (*p* < .05) and 0.3912 (*p* < .001), respectively. The NPY (Figure 8b) significantly affected soil chemical properties, physical properties, and aboveground plant biomass with pathway coefficients of 0.36, 0.31, and 0.39, respectively. Plant communities were indirectly affected by N addition and directly affected by soil physical properties, with path coefficients of 0.37 and 0.20, respectively. The plant diversity index was indirectly affected by nitrogen addition and directly affected by plant communities, with path coefficients of 0.50 and 0.05, respectively. Unlike NTY, aboveground plant biomass in NPY was significantly affected by SOC, pH, abundance, and coverage, with coefficients of −0.35, −0.34, −0.27, and 0.74, respectively, indicating that N addition directly affects aboveground plant biomass.

## 4 DISCUSSION

### 4.1 N addition is a significant factor influencing plant biomass and diversity

Plants that cannot adapt to N deposition accumulation are subject to competitive exclusion in grassland ecosystems, thereby reducing species diversity (Kinnebrew et al., 2019; Lai et al., 2018). However, our study showed that species richness and abundance did not change significantly under different N addition treatments (Figure 2a,b). The short duration of N addition in our study means its effects on plant richness and species importance values are still in their early stages (Lai et al., 2018). A 6‐year field trial demonstrated that fertilization significantly increased plant productivity but compromised plant diversity and reduced plant community stability (Band et al., 2022). Similarly, our study indicated no significant changes in plant coverage and aboveground biomass in LN and MN treatments, whereas substantial changes were observed in HN and SN compared to CK (Figure 2c,d).

Lu, Lu, et al. (2021), Lu, Hao, et al. (2021) and Lai et al. (2018) showed that long‐term excess N addition affects plant growth and diversity. In our study, NPY resulted in soil nutrient enrichment that altered soil properties (pH, salinity, and AP), affected plant growth and development, and ultimately slowly increased aboveground plant biomass (Lu, Hao, et al., 2021). In contrast, NTY satisfied plant growth and development needs rapidly increased plant productivity, reduced plant species diversity, and affected plant community stability. Thus, plant diversity showed a declining trend in NTY and an increasing trend in NPY. This indicates that the mechanism of the plant diversity index was determined by the degree of N enrichment differently between NPY and NTY. Band et al. (2022) found general agreement with our results, indicating that N‐specific mechanisms primarily affect grassland species and biomass and that N addition is the main factor influencing plant productivity and diversity.

### 4.2 Differences in soil properties due to different levels and treatments of N addition

Some studies have reported that small amounts of N stimulate plant growth and development (Tian & Niu, 2015). High N addition loads may decrease soil pH and alter plant community structure (Liu et al., 2019). Our results indicate that LN altered soil pH, whereas MN, HN, and SN did not show significant changes compared to CK (Figure 4b), which is inconsistent with Tian and Niu (2015). High soil salinity can affect plant physiological responses, nutrient and water uptake, and metabolism (Han et al., 2015). Soil moisture has been reported to promote aboveground plant biomass (Xie et al., 2015), hereby increasing soil nutrient content (Boudjabi & Chenchouni, 2022; Rao et al., 2021). Previous studies have also shown that N application can reduce the inhibition of crop enzymes by salinity (Du et al., 2022). Our study showed that N addition did not change soil water holding capacity but altered soil salt content in SN with NTY and LN with NPY. NTY significantly affected soil salinity at 0–10 cm soil depth, while NPY affected soil salinity at 10–20 cm soil depth (Figure 4c). This indicates that N addition increased salt accumulation in the plant root zone, leading to soil quality deterioration, consistent with Min et al. (2016).

N addition affects soil chemical characteristics and nutrient distribution, intrinsic indicators of soil fertility (Zhang et al., 2022). Research has shown that plant litter decomposition is the primary source of soil organic carbon (SOC) (Min et al., 2016). N addition can increase soil N content by increasing soil thickness (Chen et al., 2022). Our results showed that from 0 to 10 cm, soil TN fluctuated under NTY, with a linear decrease under NPY. Conversely, at 10–20 cm soil depth, TN showed the opposite trend (Figure 5b). Similarly, NTY SOC was significantly higher than NPY, and SOC changed considerably at different N addition levels (Figure 5a). These results are inconsistent with those of Vargas‐Lomelín et al. (2022), likely because our N addition period was only 1 year, and no long‐term stable frequency of N deposition was established. Previous studies have shown that soil N utilization and vegetation cover are higher in areas with high fertilization rates (Guo & Jiang, 2018; Sparrius & Kooijman, 2012). This variation was confirmed by our N addition experiments (Figure 2d,e). In our study, soil TP was the minimum value in HN treatment at 0–10 cm in NTY and the maximum values in SN and MN at 10–20 cm soil depth. Liu et al. (2020) showed that N addition increased C, N, and P concentrations in the subsoil and the C:P and N:P ratios. Our study found that different N addition levels and treatments were the main factors affecting soil C:N:P ecological stoichiometry ratios (Liu et al., 2020). Similarly, many studies have found that vegetation change significantly affects soil physical and chemical factors and their C:N:P ecological stoichiometric ratios (Figure 6) (Han et al., 2021).

### 4.3 Correlation analysis between plants and soil nutrients in alpine grassland

Plant diversity is significantly associated with soil spatial heterogeneity (Joshi & Garkoti, 2023; Xie et al., 2013). Meanwhile, C:P was considerably correlated with AP, indicating high phosphorus accumulation in soils (Boudjabi & Chenchouni, 2022). Figure 7a,b confirm hypothesis (1) that soil TP, TN, and AP significantly affect plant communities and diversity. This is inconsistent with Boudjabi and Chenchouni's (2022) results, as our results showed that C:P was insignificantly correlated with AP. This finding suggests that soils in the Bayinbruck alpine grasslands are less phosphorus‐limited. The significant correlation between SOC and C:N and AP suggests that SOC plays a vital role in maintaining the effectiveness of soil AP, TP, and TN in Bayinbruck alpine grassland with NTY. Liu et al. (2020) obtained results consistent with this study. The effectiveness of soil N is a predictor of plant productivity and diversity (Liu et al., 2020). Therefore, soil physical and chemical properties are essential for plant community structure and productivity (Fayiah et al., 2019).

Soil nutrient status and water availability have been shown to influence species richness (Li et al., 2024; Rodrigues et al., 2018; Tian & Niu, 2015). However, our results suggest that moisture content does not significantly affect plant community structure and productivity. Soil acidification is essential for the succession of plant communities (Han et al., 2015). Thus, pH was not the main factor affecting plant communities in this study, but salinity could indirectly affect aboveground plant biomass. Appropriate N addition can promote phosphorus uptake by plants (Zhang et al., 2022), and our study showed that AP with NPY was the main factor affecting plant community structure and diversity. Moreover, soil TN and C: N significantly affected plant diversity and aboveground plant biomass (Figure 7). In this study, soil properties were significantly correlated with plant community NTY, suggesting that N deposition affected plant diversity by reducing species richness and evenness (Yang et al., 2019; Zhou et al., 2019).

### 4.4 Pathway effects of vegetation restoration in alpine grasslands

The extent to which N addition affects soil nutrients and vegetation communities may vary according to the vegetation type or other ecosystems (Niu et al., 2018; Rodrigues et al., 2018). Generally, vegetation varies in space and time because of variations in topography, climate, weathering processes, Physico‐chemical properties of soils and microbial activities (Manral et al., 2023; Paudel & Sah, 2003) and several other biotic and abiotic factors (Pandey et al., 2024). In highly dissected landscapes, bioclimatic conditions change rapidly with the altitude and may vary within short distances resulting in a pronounced heterogeneity of soil types (Awasthi et al., 2022; Baumler, 2015), hence influencing the distribution of vegetation (Bargali et al., 2019; Manral et al., 2022). In our study, the SEM confirmed hypotheses (2) and (3) (Figure 8). As shown in Figure 8a, *R* ^2^ = 0.82 demonstrated that N addition significantly affected aboveground plant biomass and significantly changed soil properties, with an *R* ^2^ of 0.71. This indicates that the higher the level of N addition, the more significant the effect on aboveground plant biomass and soil properties. Notably, the aboveground biomass and coverage of MN, HN, and SN plants reached maximum values in NTY (Figure 2), and soil properties were significantly changed in LN and SN treatments (Figures 4 and 5). This supports the hypothesis (3) that Bayinbruck alpine grassland could be optimally restored with MN and HN treatments, while LN and SN treatments will affect grassland resilience through changes in soil AP and salinity. Similarly, in Figure 8b,n, addition significantly affected aboveground plant biomass (*R* ^2^ = 0.64). According to Figure 2d, aboveground plant biomass was significantly enhanced in the HN treatment, whereas Figures 4 and 5 show significant alterations in soil properties in LN and MN treatments. This study showed that HN among the NPY treatments had the strongest grassland restoration capacity, while changes in soil pH and SOC in LN and MN weakened grassland restoration capacity. The SEM in Figure 8b confirms Hypothesis 2 that soil pH and SOC are altered, affecting grassland resilience, with optimal restoration achieved in the HN treatment (Du et al., 2022; Lu, Lu, et al., 2021).

Fayiah et al. (2019) agreed with our study that SOC, AP, salinity, and pH play crucial roles in vegetation recovery in NTY and NPY. While their study showed that plant diversity can contribute to rapid ecosystem recovery, our study showed that increasing aboveground plant biomass can rapidly restore grassland ecosystems. Although our results might not apply to specific sites, changes in soil properties and plant communities caused by N addition may be present in many ecosystems.

## 5 CONCLUSION

This study aimed to investigate trends in plant and soil properties and the pathways of N addition for vegetation restoration in Bayinbruck alpine grasslands under different treatments and experiments. Our results showed significant alterations in plant cover and aboveground biomass under NTY and NPY. Notably, NTY slowly decreased plant diversity, while the opposite was true for NPY. Simultaneously, soil properties, especially SOC, salinity, AP, and pH, were significantly altered by N addition. According to the Mantel test and SEM, the grassland could be optimally restored with HN (15 g/m^2^) under NPY. For NTY, optimal restoration was achieved with 10 g/m^2^ and 15 g/m^2^ treatments. As N addition insignificantly changes soil properties, increasing vegetation biomass drives the most resilient grassland ecosystems. This study contributes to a better understanding of C, N, and P balances and plant community interactions in Bayinbruck alpine grasslands.

## AUTHOR CONTRIBUTIONS

**Juan Wang:** Conceptualization (equal); data curation (lead); formal analysis (equal); investigation (equal); methodology (equal); project administration (equal); resources (equal); supervision (equal); validation (equal); visualization (supporting); writing – original draft (lead); writing – review and editing (supporting). **Junjie Liu:** Funding acquisition (equal); investigation (equal); project administration (equal); supervision (supporting); writing – review and editing (supporting). **Chao Liu:** Data curation (equal); investigation (equal); resources (equal); validation (equal). **Xiaoyu Ding:** Data curation (equal); investigation (equal); resources (equal). **Yonggang Ma:** Funding acquisition (equal); investigation (equal); project administration (equal); writing – review and editing (equal). **Jianjun Yang:** Formal analysis (equal); investigation (equal); supervision (equal); writing – review and editing (equal). **Zhonglin Xu:** Funding acquisition (equal); investigation (equal); supervision (equal); writing – review and editing (equal).

## FUNDING INFORMATION

This research was funded by the Natural Science Foundation of Xinjiang Uygur Autonomous Region (No. 2023D01D01), Xinjiang University Doctoral Research Initiation Fund Project (No. 2020BS01), Talent Program (self‐taught) – “Dr. Heaven Lake” Research Program (No. tcbs201917), the Key Laboratory of Oasis Ecology (No. 2020D04003), and the Natural Science Foundation of Xinjiang Uygur Autonomous Region (Nos. 2022D01B234 and 2022E01052).

## CONFLICT OF INTEREST STATEMENT

All authors disclosed no relevant relationships.

## Supporting information


Data S1.

## Data Availability

The data that supports the findings of this study are available in the supplementary material of this article.
